# Controlling on-demand gastric acidity in obese subjects: a randomized, controlled trial comparing a single dose of 20 mg rabeprazole and 20 mg omeprazole

**DOI:** 10.1186/1471-230X-14-128

**Published:** 2014-07-15

**Authors:** Kafia Belhocine, Fabienne Vavasseur, Christelle Volteau, Laurent Flet, Yann Touchefeu, Stanislas Bruley des Varannes

**Affiliations:** 1 Institut des Maladies de l’Appareil Digestif – CHU Hôtel Dieu, 44093 Nantes Cedex, France; 2 CIC Inserm – 04, CHU Hôtel Dieu, 44093 Nantes Cedex, France; 3 Délégation à la Recherche Clinique et à l’Innovation - CHU Hôtel Dieu, 44093 Nantes Cedex, France; 4 Pharmacie, CHU Hôtel Dieu, 44093 Nantes Cedex, France; 5 UMR Inserm U913, Université de Nantes, CHU Hôtel Dieu, 44093 Nantes Cedex, France

**Keywords:** Obesity, Treatment, Pharmacology, Proton pump inhibitor, Gastroesophageal reflux disease

## Abstract

**Background:**

Obesity is associated with a risk of gastroesophageal reflux disease. The pharmacodynamic efficacy of proton pump inhibitors has not been specifically evaluated in obese subjects. The aim of this study was to compare the antisecretory response to a single oral dose of 20 mg rabeprazole, 20 mg omeprazole and placebo in obese subjects.

**Methods:**

Gastric pH was monitored for 24 hours on three separate occasions in eighteen *H. pylori*-negative, asymptomatic obese subjects. Subjects were given omeprazole, rabeprazole or placebo in a randomized order and in a double-blind fashion. The main analysis criterion was 24-h percent of time post dose with intragastric pH above 3; secondary criteria were percentage of time above pH 4, median pH, [H+] concentrations and nocturnal acid breakthrough (NAB). Results were analyzed using linear mixed models and Wilks test comparing variances.

**Results:**

24-h median [IQ] percentages of time with gastric pH above 3 and 4 were higher with rabeprazole than omeprazole (46 [37–55] vs. 30 [15–55] %, 9 [5-11] % for placebo) but the differences did not reach statistical significance (p = 0.11 and 0.24, respectively). Median acid concentrations were significantly lower with rabeprazole than with omeprazole and placebo (22 [14–53] vs. 54 [19–130] and 95 [73–170] mmoles/l, p < 0.01) for all periods. The number of NAB was significantly lower with rabeprazole than with omeprazole (median 1 [1,2] vs. 2 [1-3], p = 0.04). Variances of 24-h data (pH above 3 and 4, median pH, [H+] concentrations) were significantly lower with rabeprazole than with omeprazole (p < 0.0001).

**Conclusions:**

In asymptomatic obese subjects the gastric antisecretory response to a single dose of rabeprazole and omeprazole was strong and not significantly different between drugs despite a significantly more homogeneous response with rabeprazole.

**Trial registration:**

ClinicalTrial.gov: NCT01136317

## Background

Symptoms suggestive of gastroesophageal reflux disease (GERD) are reported by 10-20% of the population in Western countries [1]*.* Although GERD is a multifactorial disease, being overweight and obese are established as increasing the risk of developing GERD and its complications [2]. In overweight and obese subjects, direct mechanical factors and proinflammatory signals derived from the visceral adipose tissue may account for an increased occurrence of reflux episodes [3].

Acid-suppressive therapy is the main therapeutic option in GERD. However, in spite of the well-documented association between obesity and GERD, the impact of being overweight/obesity on the efficacy of acid-suppressive therapies is still poorly documented. No study has established whether high body mass index (BMI) might affect the pharmacodynamic and pharmacokinetic profile of PPIs [4]. In fact, several mechanisms such as changes in the volume of distribution of the drugs, reduction in tissue blood flow and changes in drug clearance could change the pharmacokinetics of drugs in obese patients [5-7]. PPIs as prodrugs are lipophilic compounds and may therefore have a different bioavailability in obese individuals compared to lean individuals due to variations in distribution. In addition, as most PPIs are mainly metabolized by the liver cytochrome P450 (CYP450) pathway [8], metabolism may be affected in the setting of obesity with fatty liver disease. Recently, some studies have reported a lower rate of healing of esophagitis symptoms control in patients with a high BMI treated with esomeprazole [9,10]. Likewise, Chen et al. observed that twice-daily 40 mg pantoprazole induced a better symptomatic control of reflux esophagitis in overweight/obese patients than once-daily 40 mg pantoprazole [11]. Conversely, another study using a retrospective design did not find any clear differences in mucosal healing rate between lean and overweight/obese patients treated with omeprazole or rabeprazole [12].

All these studies were conducted in patients receiving a chronic treatment on an everyday basis. In fact, a group of patients is encouraged to use PPIs on an on-demand basis adapted on the occurrence of their symptoms. Although the interest of this procedure has been established [13-15] no study has carefully determined the pharmacodynamic consequences of a such on-demand intake in obese patients. We only recently showed using a post-hoc analysis that the pharmacodynamic effects of a single dose of rabeprazole and pantoprazole were not hampered by obesity [16]. Thus, it is not known whether PPI doses should be adjusted according to body weight, especially when administered in a single dose as an on-demand treatment.

The aim of this study was to investigate prospectively the pharmacodynamic effects of single doses of 20 mg rabeprazole and 20 mg omeprazole in obese asymptomatic subjects by monitoring gastric pH for 24 hours.

## Methods

### Subjects

This single-center study was conducted at the Clinical Investigation Center of the University Hospital of Nantes (CIC INSERM-04). Eighteen ^13^C-urea breath test *Helicobacter pylori*-negative (Heli-Kit®, Mayoly Spindler, Chatou, France) obese volunteers between age 18 and 55 years were enrolled into this clinical trial.

All obese subjects had a BMI from 30 kg/m^2^ to 40 kg/m^2^ and had no clinically significant disease as determined by medical history. Subjects with clinically relevant diseases such as cardiac, renal or hepatic impairment, and patients with digestive symptoms suggestive of reflux disease or dyspepsia were not included. In addition, subjects were not included if they had known hypersensitivity to a PPI or a component of the drug, or had received acid-suppressing medications or a medication likely to interact with acid secretion in the previous month. Patients with a history of abdominal surgery were not included. During the study, reliable contraceptive methods were requested for nonmenopausal women.

Patients with consumption of alcohol more than 30 g/d or smoking more than 5 cigarettes per day were not enrolled, nor were patients with ongoing treatment by immunosuppressive therapy, antifungal or antiretroviral drugs. At the baseline enrollment, all patients underwent biological tests of liver function as well as coagulation parameters and ultrasonography of the liver to screen for any findings suggestive of nonalcoholic fatty liver disease. Also at the time of enrollment, anthropometric characteristics (weight, height, abdominal perimeter) were taken.

### Study design

This was a randomized, double-blind, placebo-controlled, three-way crossover trial. Before breakfast, subjects received one indistinguishable capsule of 20 mg rabeprazole or 20 mg omeprazole or placebo in a randomized order on three separate occasions. The marketed forms available in France, Pariet® (rabeprazole) and Mopral® (omeprazole), and the placebo (lactose tablet - Rodael laboratories - Bierne – France) were reconditioned in capsules by the central pharmacy of the hospital. Drugs were controlled and dispensed by the central pharmacy according to the randomization code for each subject. Drug administration was separated by a washout period of between 7 and 14 days.

The trial (ClinicalTrials.gov identifier: NCT01136317) was performed according to the Good Clinical Practice guidelines and in accordance with the principles for experimentation as defined in the Declaration of Helsinki. The study protocol received approval from the local Ethical Committee (Comité de Protection des Personnes, Pays de Loire N°2). All subjects received detailed written information about the trial and signed a consent form.

### Study procedure

The gastric pH monitoring procedure was performed according to the previously published procedure [17]. Briefly, all subjects were instructed to fast from 10:00 pm the night prior to their visit until their arrival at the center at 7:00 am, on each pH-monitoring day. The glass pH electrode with incorporated reference (Jubileum 1.8®, Microbioprobe and Telemedicine, Marigliano, Italy) was inserted and placed 10 cm below the esophagogastric junction as determined by the pH step-up method [18,19]*.*

The study drug was taken at 7:30 am. Twenty-four-hour ambulatory pH monitoring was then conducted from 8:00 am to 8.00 am on the following day while remaining in the center for all the entire 24 hours of recording. For the 3 occasions, subjects were given similar daily meals at the same predetermined hours (breakfast at 8:30 am, lunch at 1:00 pm and dinner at 7:00 pm), with a global intake of 1960 kcal/24 h (P:19%, L:32%, G:49%). Only still mineral water and tea were allowed without restriction. Smoking was not allowed. Subjects were required to remain in a recumbent position from 10:00 pm to 7:00 am.

Analysis was performed on the 24-h recording time, the diurnal period from 8:00 am to 10:00 pm; and the nocturnal period from 10:00 pm to 8:00 am. The primary efficacy criterion was the percentage of time with intragastric pH > 3. The secondary criteria were the percentage of time with pH > 4, median intragastric pH, acid concentration and the occurrence of nocturnal acid breakthrough (NAB). Nocturnal acid breakthrough was defined as the occurrence of intragastric pH < 4 for more than 1 h from 10:00 pm to 08:00 am [20].

### Statistics

The number of subjects to be included was calculated using the results of Williams et al’s study [21]. Using percentage of time with pH above 3 as the primary endpoint, an alpha risk of 5% and a beta risk of 20%, and considering a variation rate of 67%, 18 subjects were needed to observe a significant difference between both treatment groups.

pH-metric parameters have been expressed using medians and interquartile ranges, and percentiles if needed. Due to the crossover design of the study, mixed linear models adapted for repeated measures were used in order to compare treatment for main and secondary criteria. Treatment and period of treatment were considered as fixed effects. A lack of carryover effect as well as interaction treatment period was verified. In the model used both for main and secondary criteria, the patient was considered as a random factor. In order to test for difference in the variability of response, variances of tested parameters observed in each treatment group were compared using Wilks test [22]. The difference was considered as significant for a p value < 0.05. Analyses were performed with SAS statistical software version 9.3 (SAS Institute Inc., Cary, North Carolina).

## Results

Eighteen Caucasian subjects (36.8 ± 9.3 years, range 22–54; 9 females) completed the study and were analyzed. Median weight was 101 kg (Q1-Q3: 88–111), median BMI was 33.1 kg/m^2^ (Q1-Q3: 31.2-36.2) and median abdominal diameter was 107 cm (Q1-Q3: 107–114).

### Gastric acidity

**Figure 1:**
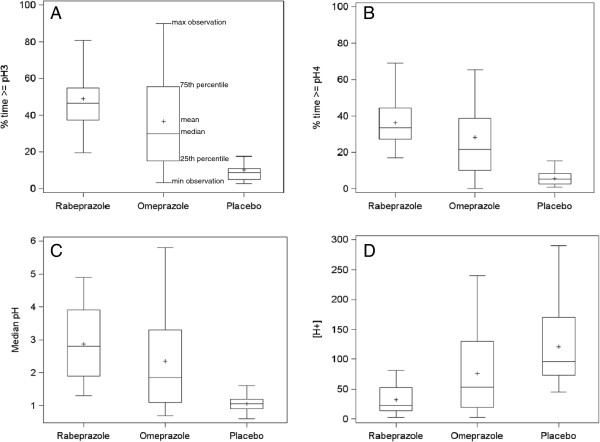
Box plots of percentage of time spent above pH 3 (A), pH 4 (B), median gastric pH (C) and median gastric acid concentration (D) in 18 obese volunteer subjects after a single oral dose (20 mg) of rabeprazole, omeprazole or placebo during 24-hour gastric pH monitoring.

The percentages of time with intragastric pH > 3 and pH > 4 are indicated in Table 1 for 24-h (Figure 1), diurnal and nocturnal periods. For percent time pH > 3 no carryover effect nor period effect was observed, but there was a significant treatment effect. The 24-h median percent time pH > 3 was higher with both PPIs as compared with the period with placebo (p = 0.0002 and p < 0.0001 for omeprazole and rabeprazole, respectively). Twenty-four-hour percent of time pH > 3 was greater with rabeprazole than with omeprazole but the difference did not reach statistical significance (p = 0.11). Regarding diurnal and nocturnal periods, percentages of time above pH 3 were significantly higher with both PPI as compared with placebo. The diurnal percentage of time above pH 3 was higher with rabeprazole than with omeprazole but the difference was not significant (p = 0.08).

**Table 1:** Percentages of time with gastric pH above 3 and 4, and gastric acid concentrations for the 24-hour, diurnal and nocturnal periods following a single dose of placebo, 20 mg omeprazole (O) or 20 mg rabeprazole (R) in 18 obese volunteers

	**Rabeprazole**	**Omeprazole**	**Placebo**	**p R vs. O**
***% time above pH3***				
• 24-hour period	46.5 (37.3-54.9)	29.7 (15.2-55.5)	8.8 (4.8-10.8)	0.10
• Diurnal period	52.1 (36.4-67.8)	32.3 (14.9-64.0)	10.2 (6.2-15.1)	0.08
• Nocturnal period	38.2 (24.2-56.6)	29.5 (6.7-49.2)	4.1 (1.9-13.2)	0.25
**% time above pH4**				
• 24-hour period	33.4 (27.2-44.3)	21.3 (10 .1-38.9)	5.3 (2.5-8.2)	0.24
• Diurnal period	37.9 (25.1-49.7)	20.1 (9.8-48.6)	4.6 (1.8-8.5)	0.21
• Nocturnal period	27.7 (14.0-49.3)	20.1 (3.3-37.4)	3.1 (0.2-10.6)	0.46
***Acid concentration [H***^***+***^ ***] mmol/l***				
• 24-hour period	22 (14–53)	54 (19–130)	95 (73–170)	0.01
• Diurnal period	27 (9–57)	53 (10–81)	97 (64–150)	0.05
• Nocturnal period	29 (18–48)	57 (23–160)	115(76–180)	0.01

Values are medians and Interquartiles (Q1-Q3).

Regarding the percentages of time with gastric pH above 4, there was a significant treatment effect for all considered periods. Although percentage of time with intragastric pH > 4 was higher with rabeprazole at any period time considered, the differences between rabeprazole and omeprazole were not statistically significant (Table 1, Figure 1). However, for any considered period, variances for time above pH 3 and pH 4 were significantly lower for rabeprazole than for omeprazole (Wilks test, p < 0.0001).

### Median gastric pH and acid concentration

**Figure 2:**
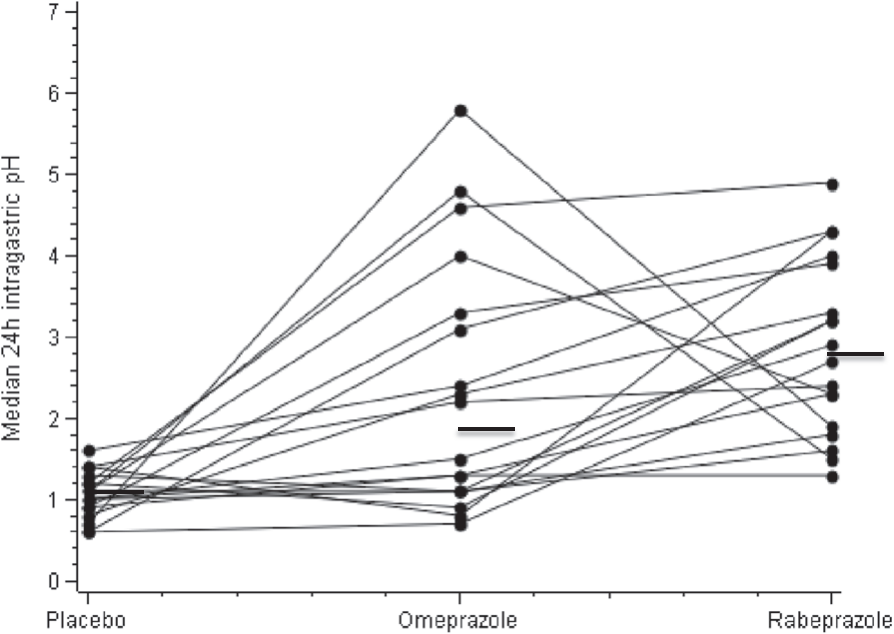
Twenty-four hour median pH individual values in 18 obese volunteer subjects after a single oral dose (20 mg) of rabeprazole, omeprazole or placebo during 24-hour gastric pH monitoring.

Twenty-four-hour individual values of median intragastric pH are shown in Figure 2. Despite higher and more homogeneous values during the rabeprazole period as compared with the omeprazole period (Figures 1 and 2) the difference did not reach statistical significance (p = 0.25). The same was true for the diurnal and nocturnal periods (p = 0.30 and p = 0.13, respectively). For the 24-hour and diurnal periods, variances for median pH were statistically lower for rabeprazole than for omeprazole (p < 0.0001).

As far as gastric acid concentrations are concerned, there was also a significant treatment effect. The median acid concentrations were significantly lower with rabeprazole than with omeprazole and placebo for the three considered analysis periods (Table 1, Figure 1). For any period, variances for acid concentrations were statistically lower for rabeprazole than for omeprazole (p < 0.0001).

### Nocturnal acid breakthrough (NAB)

The overall number of NAB (for the 18 subjects) was lower during the rabeprazole period (n = 24) than during the placebo period (n = 36) and omeprazole period (n = 37). The median occurrence of NAB per patient was low, 2 (range 1–3) both for the placebo and omeprazole periods, and 1 (range 1–2) for the rabeprazole period, with a statistically significant difference between omeprazole and rabeprazole (p = 0.04) (Table 2).

**Table 2:** Characteristics of nocturnal acid breakthrough (NAB) monitored using intragastric pH monitoring following a single dose (20 mg) of rabeprazole, omeprazole and placebo in 18 obese volunteers. Values are medians and interquartiles (Q1-Q3)

**NAB**	***Rabeprazole***	***Omeprazole***	***Placebo***	***p (OvsR)***
Number	1 (1–2)	2 (1–3)	2 (1–3)	0.04
Minimal pH	0.7 (0.6-0.9)	0.5 (0.3-0.8)	0.4 (0.3-0.6)	0.53
Median pH	1.4 (1.2-1.5)	1.3 (0.8-1.5)	0.9 (0.7-1.1)	0.47
Duration (h)	5.1 (3.4-6.2)	6.5 (4.6-8.9)	9.2 (7.9-9.9)	0.11

The median duration of NAB was shorter with both PPIs, but there was no statistical difference between the rabeprazole and omeprazole periods (p = 0.11). The nadir pH and the median pH of NAB were significantly different between the two active treatment and placebo groups (p = 0.002, p = 0.04, respectively), but there was no statistically significant difference between both drugs (Table 2). These results remained unchanged even when considering NAB on the more selective period after midnight, i.e. between 0:00 a.m. and 8:00 a.m.

### Tolerability

No serious adverse event was reported during the study. Four patients reported minor sides events likely associated with the transnasal catheter (maxillary pain, pharyngeal pain, nasal pain, epistaxis). These complaints were spontaneously resolved and the patients recovered without sequelae.

## Discussion

The results of this study show that in obese subjects a single dose of 20 mg rabeprazole and 20 mg omeprazole induced a significant reduction of gastric acidity as compared with placebo. Statistical analysis exhibited a significant treatment effect for all the analyzed criteria (percent time with intragastric pH > 3 and 4 and median pH). As far as the main endpoint was considered (percent time with intragastric pH > 3), there was no statistically significant difference between both drugs. However, there was a statistically significant decrease (approximately 50%) in the median gastric acid concentration after administration of 20 mg rabeprazole vs. 20 mg omeprazole (Table 1, Figure 1). Of interest is also the efficiency of rabeprazole in significantly reducing the occurrence of NAB. Reduced NAB episodes have previously been reported even when using low doses of rabeprazole (10 mg) in comparison with a standard dose of omeprazole (20 mg) or pantoprazole (40 mg) [23].

Previous studies in healthy, nonobese, *H.pylori*-negative subjects using similar methodology to compare the antisecretory effects of single doses of various PPIs documented similar findings of increased mean intragastric pH, increased percentage of time with pH > 3 or > 4, and decreased intragastric [H+] with rabeprazole compared with omeprazole, lanzoprazole or pantoprazole [24,25]. Regarding omeprazole, and although the omeprazole dose (i.e. 20 mg daily) is the approved daily dose in the treatment of GERD, several pharmacodynamics studies have shown that it is not equivalent to the approved regimens of the other PPIs (namely lansoprazole, pantoprazole and rabeprazole). In addition, the inter-individual variability of the antisecretory action of the 20 mg dose of omeprazole has been well described from its introduction in our therapeutic armamentarium [26,27]. In our study, the lack of demonstration of a statistically significant difference between omeprazole and rabeprazole for the main endpoint, i.e. 24-h percent time pH > 3, may be due to a second espece risk. This difference may also suggest that in obese patients the overall antisecretory difference between PPIs is less than that observed in nonobese subjects or patients.

The pharmacokinetics of drugs may be affected by obesity due to alterations in drug distribution mediated by reduced tissue blood flow [6]. In addition, drug clearance may be affected in obese patients as a result of changes in renal and hepatic physiology [4]. The present study does not explain the causes of observed differences between the effects of the two PPIs in obese subjects. Previous evidence of impaired cytochrome P450 metabolism reported in obesity [28] may partially account for the observed advantage of rabeprazole over omeprazole due to increased metabolism of rabeprazole by nonenzymatic pathways and decreased dependency on CYP2C [29]. In fact, the influence of obesity on CYP450 appears to be isoenzyme specific, with a decrease of CYP3A4 activity and an increase of CYP2E1.

As illustrated by the significantly lower variances for time above pH3 and pH4 and median pH and acid concentrations, the decreased variability of antisecretory response with single-dose rabeprazole in obese subjects may represent an advantage when using PPI intermittently on demand in response to insufficiently controlled symptoms of GERD before suggesting a continuous treatment. Further studies in obese subjects are needed to determine whether these pharmacodynamic differences between rabeprazole and omeprazole persist when taking the drug consistently on a daily basis, as has previously been documented in nonobese subjects [21].

Our study has several strengths. It is one of the first pharmacodynamic studies specifically conducted in obese subjects. Obese asymptomatic subjects were well characterized in order to exclude any hepatic abnormality, and pharmacodynamic data were obtained in rigorous conditions using continuous monitoring of gastric pH. Our study also has some limitations including small sample size and the lack of a nonobese control group. In fact, the final word on the effect of obesity on PPI pharmacokinetics and pharmacodynamics will be given by a study, investigating both activities of a given PPI before and after weight reduction, whether obtained by medical or surgical treatment.

## Conclusions

In conclusion, this study shows that in obese healthy volunteers the overall antisecretory effect after a single dose of 20 mg rabeprazole and 20 mg omeprazole, when taken on an on-demand basis, is strong but not significantly different between both drugs. However, the antisecretory response seems to be more homogeneous after rabeprazole. Finally, our results suggest that, from a pharmacodynamic point of view, dose augmentation in obese subjects is not justified.

## Abbreviations

BMI: Body mass index; GERD: Gastroesophageal reflux disease; NAB: Nocturnal acid breakthrough; PPI: Proton pump inhibitor.

## Competing interests

K. Belhocine, F. Vavasseur, C. Volteau, L Flet, Y Touchefeu: none; S. Bruley des Varannes has served as a speaker, consultant and/or advisory board member for Given Imaging, Cephalon, Mayoly Spindler, Janssen, Alfa Wassermann, Almirall.

## Authors’ contributions

KB principal investigator, volunteer selection, subject-related study procedures, data analysis, manuscript writing; FV investigator, volunteer selection, subject-related study procedures; CV data analysis, statistical analysis; LF drug packaging, subject-related study procedures; YT data analysis, manuscript writing; SBV protocol design, principal investigator, volunteer selection, data analysis, manuscript writing. All authors have read and approved the final manuscript.

## Pre-publication history

The pre-publication history for this paper can be accessed here:

http://www.biomedcentral.com/1471-230X/14/128/prepub
